# Microbial responses to southward and northward Cambisol soil transplant

**DOI:** 10.1002/mbo3.302

**Published:** 2015-10-26

**Authors:** Mengmeng Wang, Shanshan Liu, Feng Wang, Bo Sun, Jizhong Zhou, Yunfeng Yang

**Affiliations:** ^1^ State Key Joint Laboratory of Environment Simulation and Pollution ControlSchool of EnvironmentTsinghua UniversityBeijing100084China; ^2^ Collaborative Innovation Center for Regional Environmental QualityTsinghua UniversityBeijing100084China; ^3^ State Key Laboratory of Soil and Sustainable AgricultureInstitute of Soil ScienceChinese Academy of SciencesNanjing210008China; ^4^ Ningbo Academy of Agricultural SciencesNingbo315040China; ^5^ Institute for Environmental Genomics and Department Microbiology and Plant ScienceUniversity of OklahomaNormanOklahoma73019; ^6^ Earth Sciences DivisionLawrence Berkeley National LaboratoryBerkeleyCalifornia94720

**Keywords:** Cambisol soil, microbial diversity, microbial functional potential, soil transplant

## Abstract

Soil transplant serves as a proxy to simulate climate changes. Recently, we have shown that southward transplant of black soil and northward transplant of red soil altered soil microbial communities and biogeochemical variables. However, fundamental differences in soil types have prevented direct comparison between southward and northward transplants. To tackle it, herein we report an analysis of microbial communities of Cambisol soil in an agriculture field after 4 years of adaptation to southward and northward soil transplants over large transects. Analysis of bare fallow soils revealed concurrent increase in microbial functional diversity and coarse‐scale taxonomic diversity at both transplanted sites, as detected by GeoChip 3.0 and DGGE, respectively. Furthermore, a correlation between microbial functional diversity and taxonomic diversity was detected, which was masked in maize cropped soils. Mean annual temperature, soil moisture, and nitrate (NO_3_¯‐N) showed strong correlations with microbial communities. In addition, abundances of ammonium‐oxidizing genes (*amoA*) and denitrification genes were correlated with nitrification capacity and NO_3_¯‐N contents, suggesting that microbial responses to soil transplant could alter microbe‐mediated biogeochemical cycle at the ecosystem level.

## Introduction

Extreme climate change events, such as abrupt warming or cooling, are occurring with elevated frequency and/or intensity (Alley et al. 2003; Rahmstorf and Coumou 2011), which impose substantial impacts on terrestrial ecosystems. Since microbial communities are essential in mediating soil biogeochemical cycle (Zhou et al. 2012; Chu et al. 2014), it is imperative to elucidate microbial responses to climate changes. Soil transplant, which exposes soil to realistic climate regimes at different places, has recently been established as an experimental strategy to simulate climate changes (Balser and Firestone 2005; Breeuwer et al. 2010; De Frenne et al. 2011; Lazzaro et al. 2011; Vanhala et al. 2011). Using this strategy, we have recently shown that both southward transplant of black soil (typical of cold temperate climate zone) and northward transplant of red soil (typical of subtropical and tropical climate zone) altered microbial community structure and soil biogeochemical cycles (Zhao et al. 2014a; Liu et al. 2015). However, soil type is a fundamental factor in shaping microbial communities (Schimel and Chadwick 2013), rendering it unlikely to compare across different soil types. Therefore, it remains unclear how microbial communities respond to soil transplant toward converse perturbation.

In this study, we analyzed Cambisol soil subjected to southward and northward transplant over large transects. With an area of 35 million hectares, Cambisol soil is the main soil type in North China Plain, one of the most important agricultural regions in China (Ding et al. 2010). Since vegetation has been shown to have an interactive effect with soil transplant on microbial communities (Zhao et al. 2014a; Liu et al. 2015). In this study, we analyzed both bare fallow and maize cropped soils, with a focus on bare fallow soil. We specifically addressed the following scientific questions: How did soil transplant and vegetation alter microbial communities and soil biogeochemical cycles? Are there remarkable linkages between microbial communities and environmental variables? Are climatic variables important in influencing microbial communities? We used a metagenomic tool named GeoChip 3.0 to analyze microbial functional potentials, denaturing gradient gel electrophoresis (DGGE) to analyze coarse‐scale taxonomy, and phospholipid fatty acids (PLFA) to analyze microbial biomass.

## Materials and Methods

### Sites and sampling

This study was performed in three experimental stations managed by Chinese Academy of Science: Fengqiu station (35°00′ N and 114°24′ E, warm temperate climate zone) of Henan Province in Central China, Yingtan station (28°15′ N and 116°55′ E, subtropical climate zone) of Jiangxi Province in Southern China and Hailun station (47°26′ N and 126°38′E, cold temperate climate zone) of Heilongjiang Province in Northern China. Herein we designate these stations as C, S, and N according to their geographical locations. The distance between C and S is 784 km, and the distance between C and N is 1398 km. Their climatic variables are summarized in Table 1. The soil type at the C site is fluvo‐aquic soil, which belongs to Cambisol in the FAO soil classification system (IUSS Working Group WRB, 2006) (Qian et al. 2013). The soil was derived from alluvial sediments of the Yellow River and has a sandy loam texture (72% silt, 17% sand, and 11% clay) (Ding et al. 2010).

**Table 1:** Summary of environmental variables

	CC	CN	CS
Climate variables			
Mean annual temperature (MAT, °C)	13.80	1.60	18.40
Mean annual precipitation (MAP, mm)	387.00	430.00	1369.00
Relative humidity (RH, %)	71.83	70.51	79.58
Soil variables			
pH	7.70 ± 0.12 a^a^	7.68 ± 0.14 a	7.24 ± 0.47 a
Soil moisture (%)	**5.91 ± 0.57 c**	**7.86 ± 0.60 b**	**19.20 ± 0.20 a**
Soil temperature (°C)	**25.81 ± 0.10 b**	**24.83 ± 0.03 c**	**32.92 ± 0.18 a**
Organic matter (g/kg)	9.49 ± 0.32 a	9.57 ± 0.21 a	8.58 ± 0.63 a
Nitrate (NO_3_¯‐N, mg/kg)	**32.88 ± 10.38 a**	**14.28 ± 2.92 ab**	**7.84 ± 4.24 b**
Ammonium (*NH*_4_^+^‐N, mg/kg)	1.70 ± 0.07 a	1.51 ± 0.16 a	1.64 ± 0.06 a
Total nitrogen (TN, g/kg)	0.61 ± 0.06 a	0.60 ± 0.02 a	0.57 ± 0.04 a
Total phosphorus (TP, g/kg)	0.62 ± 0.02 a	0.57 ± 0.02 a	0.56 ± 0.06 a
Total potassium (TK, g/kg)	17.84 ± 0.17 a	17.19 ± 0.10 a	14.68 ± 2.66 a
Alkali‐hydrolyzable nitrogen (AN, mg/kg)	41.81 ± 1.10 a	40.71 ± 1.10 a	45.11 ± 3.97 a
Available phosphorus (AP, mg/kg)	6.58 ± 0.26 a	6.60 ± 1.04 a	11.53 ± 3.80 a
Available potassium (AK, mg/kg)	95.83 ± 5.07 a	115.00 ± 10.00 a	95.00 ± 18.87 a
Soil biological process			
Nitrification capacity (%)	**14.84 ± 5.45 b**	**38.72 ± 4.36 a**	**25.01 ± 3.91 ab**

a Results are presented as mean ± standard error (SE, *n* = 3). Significance was tested by one‐way ANOVA. Significantly changed variables (*P *<* *0.10) are marked in bold.

In October 2005, six replicate plots of soil with the size of 1.4 m × 1.2 m × 1.0 m (length × width × depth) were transplanted from C to S and another six replicates were transplanted to N. The transplanted samples were thereafter designated as CS and CN, respectively. As controls, six replicate plots of the same size at C, S, and N sites were treated with the same procedure but mock transplanted in place, which were named as CC, SS, and NN, respectively. To minimize perturbation on soil structure, soil was excavated in five layers of 20 cm depth, transplanted to destination plots, and placed in the same order of layers as original soil. The plots were separated from neighboring, native soil by a surrounding 20‐cm brick wall and waterproof cloth and underneath quartz sand. Maize was planted in three plots of each six plots every year since 2006, and samples with maize cropping were designated as CSm, CNm, CCm, NNm, or SSm. In plots without maize cropping, soils were kept bare by manual weeding.

After 4 year of adaptation at the transplanted sites, soil samples were collected between late August and early September of 2009. At each plot, 10 soil cores with a diameter of 2 cm were taken at the depth of 0–20 cm and composited into a soil sample. The soil was then sealed into a polythene wrap and transported to the laboratory, where it was divided into two subsamples. One subsample was stored at 4°C for soil variable measurements and the other was stored at −80°C for microbial DNA analyses.

### Measurements of environmental variables

Soil moisture was measured gravimetrically after overdrying at 105°C for 12 h. Soil pH was measured using a glass electrode (Mettler Toledo Instruments, Shanghai, China) in a dilution of soil (soil:water ratio of 1:2.5). Soil organic matter was measured by titration with ferrous ammonium sulfate after dichromate oxidation. Measurements of total nitrogen (TN) and alkali‐hydrolyzable nitrogen (AN) used Kjeldahl digestion and the Illinois Soil Nitrogen Test (ISNT) diffusion method, respectively. Concentrations of nitrate ( ${\text{NO}}_{3}^{-}$‐N) and ammonium ( ${\text{NH}}_{4}^{+}$‐N) were measured by Auto Analyser 3 (Bran+Luebbe GmbH, Germany) in a suspension of soil and 1 mol/L KCl (soil:KCl ratio of 1:5). Total phosphorous (TP) and available phosphorous (AP) were extracted by sodium carbonate and sodium bicarbonate, respectively, and measured by a molybdenum blue method. Total potassium (TK) and available potassium (AK) were fused with sodium hydroxide and extracted by ammonium acetate, respectively, and measured with a flame photometry (FP66400A, CANY Precision Instrument Co., Ltd., Shanghai, China).

Soil nitrification capacity was measured as described previously (Liu et al. 2015). Briefly, a total of 31.5 mg (NH_4_)_2_SO_4_ was added to 70 g of soil (oven‐dry weight equivalent), and soil moisture was adjusted to 70% of water holding capacity by adding water. And then soil was mixed well and incubated in the dark at 25°C for 14 days. On Days 0, 2, 3, 7, 10, and 13, concentrations of ${\text{NH}}_{4}^{+}$ ${\text{NO}}_{2}^{-}$ , and ${\text{NO}}_{3}^{-}$ were measured by a continuous flow analyzer (Bran+Luebbe GmbH Inc., Germany) in 1 mol/L KCl extractant (soil:KCl ratio of 1:5). Climatic variables of mean annual temperature (MAT), mean annual precipitation (MAP), and relative humidity were obtained from the records of local meteorological stations.

### Analyses of soil microbial communities

Phospholipid fatty acid (PLFA) of microbial communities was extracted using a modified procedure as described previously (Brant et al. 2006). Briefly, 2 g of dry soil was mixed with a solution composed of methanol, chloroform, and phosphate buffer in the ratio of 2:1:0.8, followed by incubation for 2 h. Phospholipids were separated from extracted lipids using silica acid columns. The phospholipids were then transferred to fatty acid methyl esters (FAME) and analyzed by a Sherlock Microbial Identification System (MIDI Inc., Newark, DE). PLFAs of bacteria, fungi, and actinomycetes were discerned using established markers (Mikola and Setälä 1999).

For denaturing gradient gel electrophoresis (DGGE) analysis, DNA was extracted by a FastDNA^®^ SPIN Kit (MP Biomedicals, Santa Ana, CA) according to the manufacturer's instructions. The V3 region of 16S rRNA was amplified by primers F338 and R541 as described previously (Muyzer et al. 1993). After electrophoresis on a CBS DGGE 2000 system (C.B.S. Scientific Co., Inc., Del Mar, CA), DNA amplicons were stained with SYBR green I dye (Cambrex Bioscience, Walkersville, MD) and quantified with an imaging system Bio‐Rad Molecular Imager Gel Doc XR (Bio‐Rad Laboratories Inc., Hercules, CA).

For GeoChip analysis, DNA was extracted by a freeze–grinding method as described previously (Zhou et al. 1996), and it was then purified in agarose gel electrophoresis. The excised gel slice was melted, extracted, and purified in sequential steps with an equal volume of cold, buffer‐saturated phenol and then phenol–chloroform (in the ratio of 1:24). DNA was precipitated by adding 10 volumes of 3 mol/L NaOAc (pH = 5.2) and two volumes of cold 100% ethanol. DNA quality was evaluated by the ratios of A_260_/A_280_ and A_260_/A_230_ using a Nanodrop (NanoDrop Technologies Inc., Wilmington, DE) and its concentration was measured by the PicoGreen method (Ahn et al. 1996).

GeoChip 3.0 experiments were performed as described previously (Zhao et al. 2014b). In brief, 2 *μ*g of purified DNA was labeled with Cy‐5 fluorescent dye and then hybridized with GeoChip 3.0 at 42°C for 16 h. After washing away unbounded DNA, microarrays were scanned with a ProScan Array scanner (Perkin Elmer, Waltham, MA) and the signal intensity of each probe was measured by an Imagene 6.0 software (Biodiscovery, El Segundo, CA).

### Data analyses

Raw data of GeoChip were analyzed as described previously (Ding et al. 2015). In brief, the following steps were performed: (1) spots flagged or with a signal‐to‐noise ratio (SNR) <2.0 were considered as poor‐quality spots and thus removed; (2) probes detected only once among three replicates were considered as possibly false positives and thus removed; and (3) the intensity of each spot was transformed by natural logarithm and then normalized by dividing the average intensity of the microarray.

The microbial functional diversity and coarse‐scale taxonomic alpha diversity were calculated based on GeoChip data and band patterns and intensity of DGGE, respectively, using Shannon index. Environmental variables were tested for significant differences using a one‐way analysis of variance (ANOVA). Detrended correspondence analysis (DCA) was used to examine the overall change in microbial communities. Dissimilarity test of adonis was performed to verify results of DCA. Multiple regression trees (MRT) were used to analyze complex ecological data to determine high‐order interactions by forming clusters with minimum dissimilarity based on environmental characteristics (De'Ath 2002). Partial Mantel tests and canonical correspondence analysis (CCA) were used to calculate correlations between microbial community and environmental variables. For CCA modeling, variables showing variance inflation factor (VIF) >20 was removed to prevent covariation. Distance matrices of microbial communities and environmental variables were calculated using the Euclidean method. Pearson correlation tests were performed to examine if there were linear relationships between two datasets. These data analyses were performed by using the functions in the Vegan package 2.0–8 in R 3.0.1 (Oksanen et al. 2007).

## Results

### Changes in edaphic variables and microbial communities by soil transplant

Soil samples were collected in the late summer of 2009. The *in situ* soil temperature of CN and CS samples was −1.0°C lower and 7.1°C higher than that of CC, respectively (Table 1). Soil moisture was increased by 32.9% in CN samples and 224.9% in CS samples. Soil nitrate (NO_3_¯‐N) contents were significantly decreased by 76.2% in CS samples and remained unchanged in CN samples, while total N, ammonium (NH_4_^+^‐N), organic matter, total phosphorus, total potassium, alkali‐hydrolyzable nitrogen, available phosphorus, and available potassium contents remained largely unchanged.

**Figure 1:**
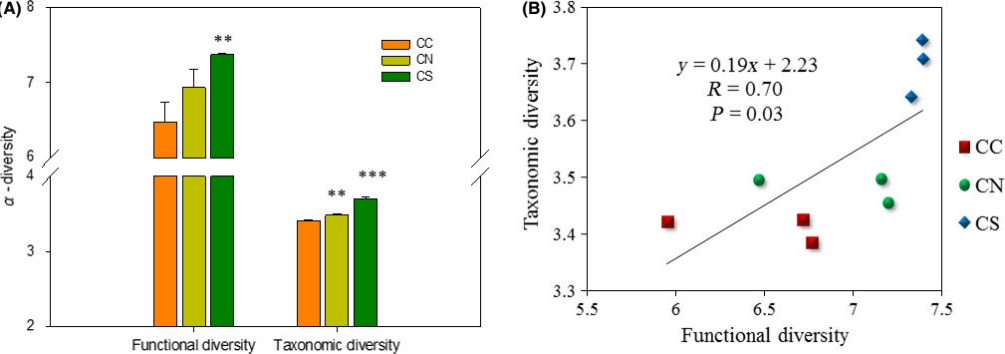
(A) The effect of soil transplant on microbial functional and coarse‐scale taxonomic alpha diversity in bare fallow soils. The functional diversity and coarse‐scale taxonomic alpha diversity were calculated based on GeoChip data and denaturing gradient gel electrophoresis (DGGE) band patterns and intensities, using the Shannon index. Error bars represent standard errors (SE,* n* = 3). Significant differences between CC and CN, or between CC and CS, were tested by two‐tailed *t*‐tests with equal variances, as indicated by **P <* *0.05 or ***P <* *0.01. (B) Pearson correlation between microbial functional diversity and coarse‐scale taxonomic alpha diversity.

Soil microbial communities were examined by GeoChip and DGGE to profile microbial functional potentials and coarse‐scale taxonomic diversity, respectively. Interestingly, both functional and taxonomic diversity were increased in CS and CN samples (Fig. 1A). Furthermore, a strong, positive Pearson correlation was detected between functional and taxonomic diversity (*R* = 0.70, *P *=* *0.03, Fig. 1B), suggesting that species diversification enhanced microbial functional potentials. PLFA analyses indicated that total, bacterial and fungal biomass were increased in CS and CN samples (Fig. S1), but they were neither correlated with functional diversity nor with taxonomic diversity (*R* < 0.41, *P *>* *0.27). Ratios of fungal‐to‐bacterial biomass remained unchanged (0.123 ± 0.004 for CC, 0.127 ± 0.004 for CN, and 0.178 ± 0.055 for CS).

**Figure 2:**
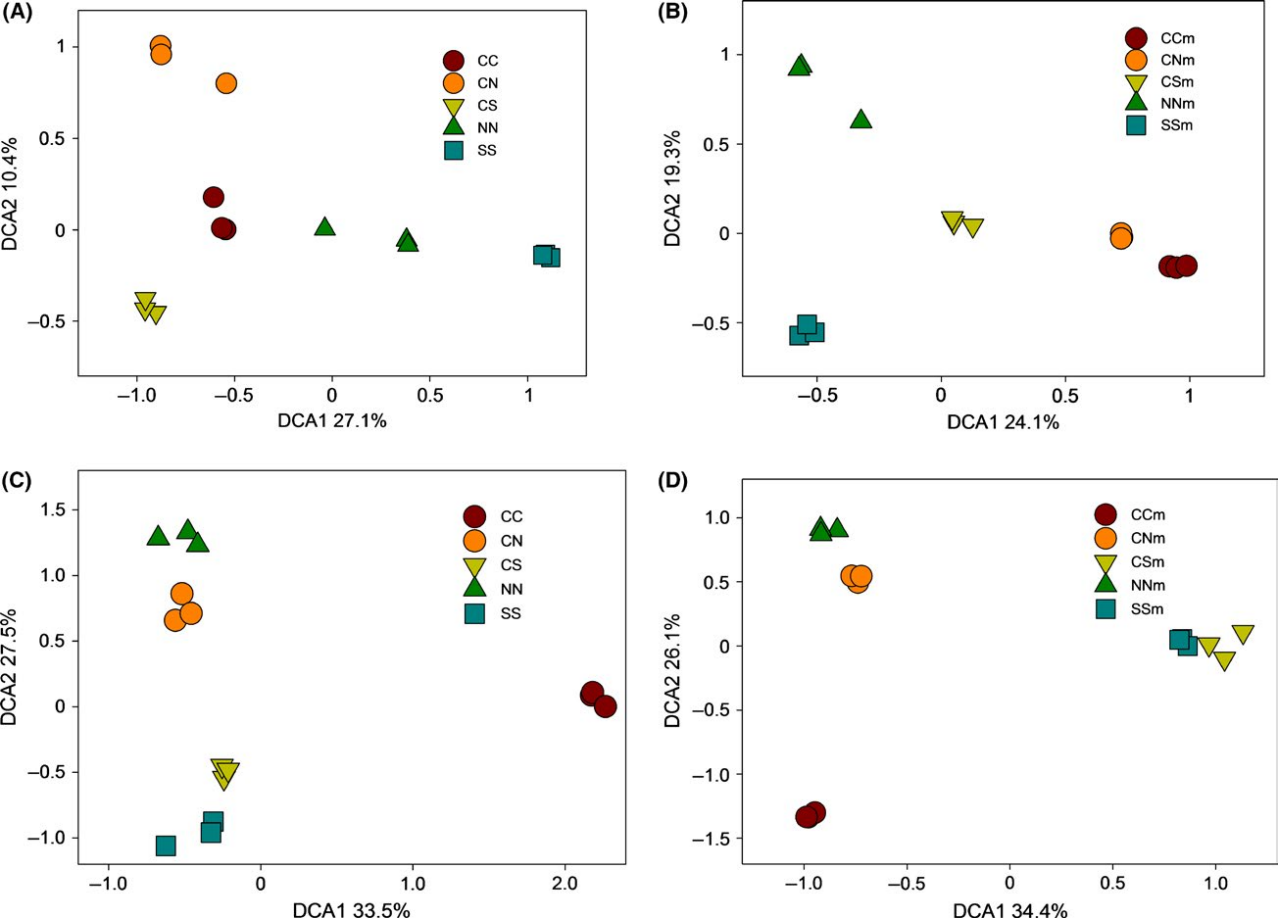
Detrended correspondence analysis (DCA) analyses of (A) GeoChip data in bare fallow soil, (B) GeoChip data in maize cropped soil, (C) denaturing gradient gel electrophoresis (DGGE) data in bare fallow soil, and (D) DGGE data in maize cropped soil.

Microbial community compositions were altered by soil transplant, as indicated by detrended correspondence analysis (DCA) of GeoChip and DGGE data (Fig. 2). To verify this, a dissimilarity test of *adonis* was carried out, which showed that differences in microbial community compositions were significant (*P *<* *0.05) or marginally significant (*P *<* *0.1) (Table S1). To exclude the possibility that changes in microbial community were caused by microbial invasion from neighboring, native soil of the transplanted sites, we took a closer examination of microbial community compositions. We found that transplanted soil shared a much higher percentage with CC soil than neighboring soil (Fig. S2), suggesting that invasion of neighboring soil, if any, was minor.

### Effect of maize cropping on microbial communities

For soils with maize cropping, both northward and southward transplants decreased functional diversity, but northward transplant increased taxonomic diversity (Fig. S3A). In addition, microbial functional and taxonomic diversity were not correlated in cropped soils (Fig. S3B, *R* = −0.32, *P *=* *0.39). MRT analysis split the GeoChip data into two clusters with or without maize cropping, suggesting that the effect of maize cropping overrode that of soil transplant (Fig. S4). To focus on soil transplant, we analyzed only bare fallow soils in the following sections.

### Major environmental variables linking to microbial communities

**Figure 3:**
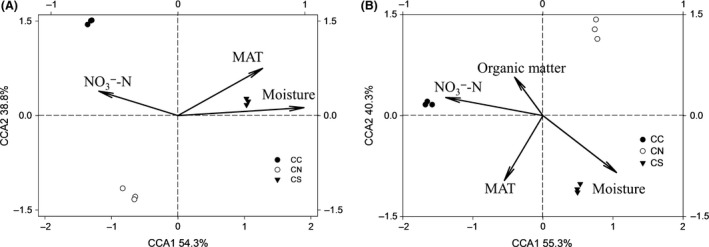
Canonical correspondence analysis (CCA) between (A) GeoChip data, (B) denaturing gradient gel electrophoresis (DGGE) data and environmental variables. MAT, mean annual temperature.

We assessed whether there were linkages between microbial communities and environmental variables. To this end, canonical correspondence analysis (CCA) and partial Mantel tests were carried out. Significant CCA models were generated for GeoChip and DGGE (*P *=* *0.008 and 0.005, respectively, Fig. 3). Among them, MAT, soil moisture, and NO_3_¯‐N showed strong correlations with microbial communities. Partial Mantel tests revealed that soil moisture showed the strongest correlations with functional compositions of microbial community, while MAT showed the strongest correlations with taxonomic compositions (Table S2). In addition, strong, positive Pearson correlations between microbial community and environmental variables were observed (*P *=* *0.01, Fig. S5).

### Soil nitrogen cycle linking to microbial functional potential genes of nitrogen cycle

**Figure 4:**
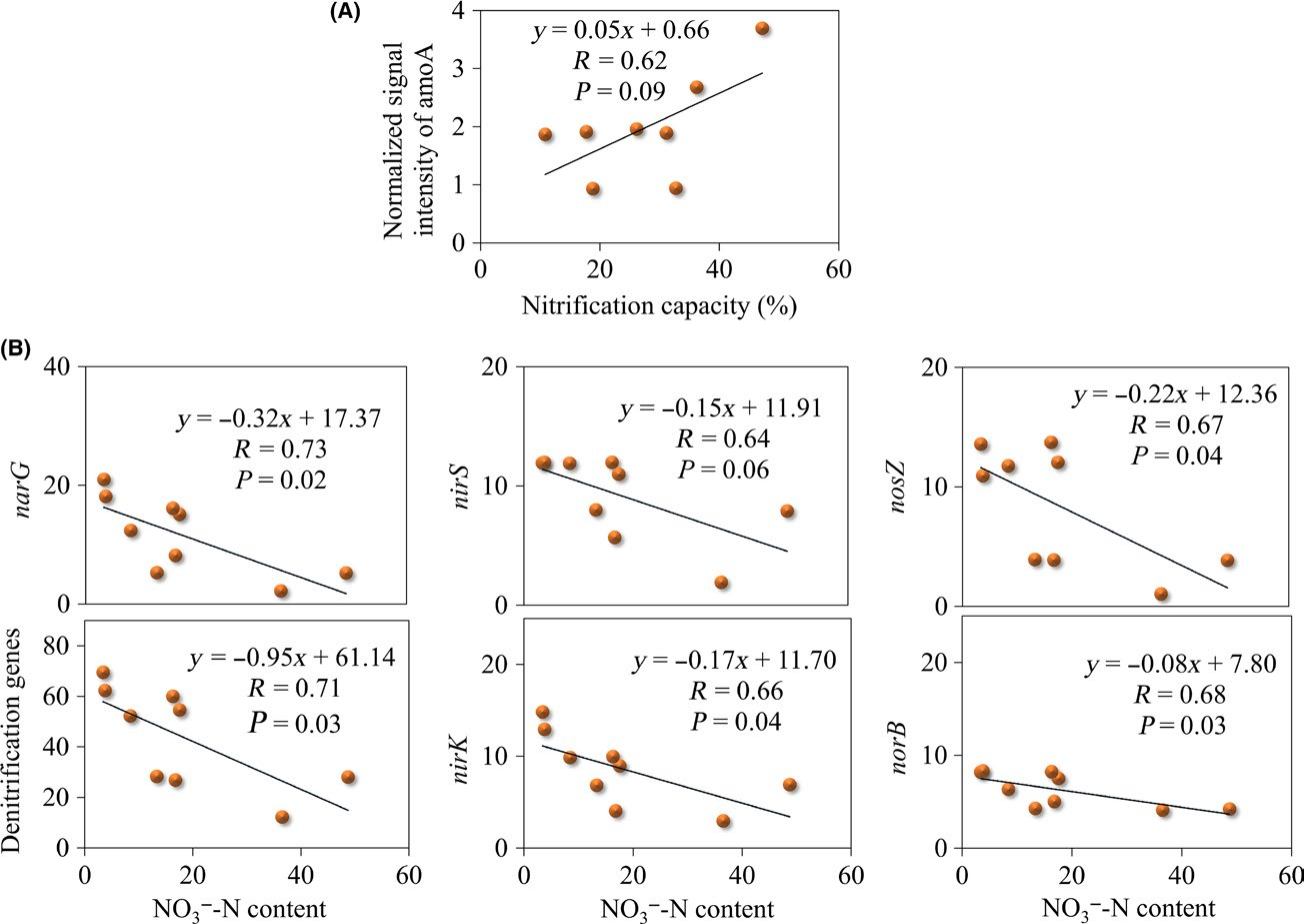
Pearson correlation between (A) normalized signal intensity of *amoA* gene and nitrification capacity; (B) normalized signal intensities of denitrification genes and NO_3_¯${\text{NO}}_{3}^{-}$‐N contents.

Nitrification capacity was significantly increased by 161.0% in CS samples and remained unchanged in CN samples (Table 1). There was a modest, marginally significant correlation between the abundance of *amoA* gene and nitrification capacity (*R* = 0.62, *P *=* *0.09, Fig. 4A). In addition, abundances of denitrification genes *narG*,* nirS*,* nirK*,* norB*, and *nosZ* were substantially increased in CN and CS samples (Fig. S6), which provided an explanation to the decrease of NO_3_¯‐N contents in the transplanted samples. Modest, negative correlations were shown between NO_3_¯‐N contents and abundances of these denitrification genes (*R* > 0.64, *P *<* *0.06, Fig. 4B).

## Discussion

In this study, we found that soil transplant and maize cropping had interactive effects on microbial communities (Figs. S3 and S4). We examined soil transplant by focusing on bare fallow soils, which was common in agricultural fields. It was interesting to note that microbial functional and taxonomic diversity were increased by both northward and southward soil transplants (Fig. 1), which was at odds with our previous finding that northward transplant of red soil decreased functional diversity (Liu et al. 2015). As we have excluded the possibility of substantial invasion from neighboring soil (Fig. S2), environmental changes at the transplanted sites could provide a likely explanation. Both temperature and water availability can change microbial communities through physiological acclimation or evolutionary adaptation (Schimel et al. 2007; Sheik et al. 2011; Sistla et al. 2013). Although a global survey of planktonic marine bacteria along a latitudinal diversity gradient showed that temperature had a positive linkage to microbial diversity (Fuhrman et al. 2008), temperature was unlikely to play a predominant role in this study since northward transplant to a cooler region increased microbial functional and taxonomic diversity (Fig. 1). Rather, water availability might be a key in shaping microbial communities. It was noted that mean annual precipitation (MAP) was increased from 387 to 430 mm by northward transplant and to 1369 mm by southward transplant (Table 1). Soil moisture was also increased and significantly (*P *<* *0.04) correlated with both taxonomic and functional compositions of microbial communities, as shown by partial Mantel tests (Table S2). It has been reported that precipitation might have a greater effect than temperature on microbial evolutionary adaptation (Castro et al. 2010). The importance of historic precipitation events was also demonstrated by the recent finding that microbial communities were sensitive to historical precipitation regimes (Evans and Wallenstein 2011). Meanwhile, changes in climate regimes might indirectly affect microbial community through changes in soil geochemical variables (Fierer and Jackson 2006; Chu et al. 2014; Yang et al. 2014). For example, nitrate contents were decreased from 32.88 ± 10.38 mg/kg to 14.28 ± 2.92 mg/kg by northward transplant and to 7.84 ± 4.24 mg/kg by southward transplant (Table 1), and nitrate contents were significantly (*P *<* *0.05) correlated with taxonomic compositions of microbial communities (Table S2). Furthermore, dissimilarity of microbial functional compositions was correlated with that of environmental variables (Fig. S5), suggesting that environmental variables contributed significantly to microbial communities.

It has been proposed that microbial communities can be altered by environmental changes but owing to functional redundancy, changes in microbial community compositions may not be reflected in the ecosystem‐level functional processes (Allison and Martiny 2008; Wall et al. 2013). However, a recent parallel 16s rRNA gene and shotgun sequencing study of microbial communities in tallgrass prairie soils showed a strong positive correlation between taxonomic and functional diversity, which was also observed in plants and animals (Petchey and Gaston 2002; Devictor et al. 2010) and suggested that functional redundancy within microbial community might be lower than commonly believed (Fierer et al. 2013). Our finding of correlation between taxonomic and functional diversity (Fig. 1B) provides additional support to this observation. Furthermore, it justifies the use of functional diversity measured by GeoChip as an additional diversity index for biodiversity assessments. It can thus be predicted that reduction in the taxonomic diversity is associated with the decrease in functional potential.

Change in microbial communities by soil transplant could lead to alteration in microbial functional potential capacities, which could consequently change ecosystem‐level functional processes (Zhao et al. 2014a; Liu et al. 2015). Abundance of *amoA* genes and nitrification capacity were strongly correlated with soil moisture (Emmett et al. 2004; Horz et al. 2004). Increased soil moisture can enhance nitrification but very high soil moisture can inhibit nitrification by limiting oxygen diffusion. In our study, both northward and southward transplants increased soil moisture (Table 1), and nitrification capacity and abundances of *amoA* were also increased (Table 1 and Fig. S6). Accordingly, abundances of *amoA* were positively correlated with soil nitrification capacity (Fig. 4A). In addition, denitrification genes had modest, negative correlations with NO_3_¯‐N contents (Fig. 4B), possibly owing to loss of soil nitrogen via N_2_O and N_2_ emissions. These findings demonstrated that changes in microbial functional potentials provided sound explanation to soil biogeochemical cycles. Therefore, it is possible to monitor functional gene abundances for assessing greenhouse gas emissions and activities of functional processes (Morales et al. 2010; Yue et al. 2015).

In consistency with recent studies showing that warming could stimulate carbon degradation (Zhou et al. 2012; Li et al. 2013), southward transplant appeared to stimulate carbon degradation since abundances of most carbon degradation genes were increased, including *XylA*,* cellobiase*, and *exoglucanase* genes associated with degradation of hemicellulose and cellulose, and *LimEH*,* glx*, and *lip* genes associated with degradation of aromatics and lignin (Fig. S7). This observation was concomitant with decreased organic matter contents in CS samples (Table 1). In contrast, few carbon degradation genes were significantly changed by northward transplant. Accordingly, organic matter contents remained unaltered by northward transplant.

In summary, here we report an integrated analysis to dissect microbial responses to southward and northward soil transplant. Both microbial taxonomic and functional diversity were increased at the transplanted sites. The linkages between microbial functional potential genes and microbially mediated eco‐processes enable a more accurate projection of soil biogeochemical cycle based on microbial functional potentials.

## Data Accessibility

GeoChip data are available online (http://www.ncbi.nlm.nih.gov/geo/) with the accession number GSE51592.

## Conflict of Interest

None declared.

## Supporting information


**Table S1.** The effect of soil transplant on functional and taxonomic community composition revealed by the dissimilarity test of *adonis* ^a^.
**Table S2.** Partial Mantel tests linking environmental variables to GeoChip and DGGE data.
**Figure S1.** Microbial biomass measured by the phospholipid fatty acid (PLFA) analysis.
**Figure S2.** Microbial composition of (A) CN and (B) CS samples revealed by Venn diagrams.
**Figure S3.** (A) The effect of soil transplant on microbial functional and coarse‐scale taxonomic alpha diversity in maize cropped soil.
**Figure S4.** Multiple regression tree analysis of GeoChip data.
**Figure S5.** Pearson correlation between dissimilarity of microbial functional compositions and environmental variables.
**Figure S6.** Impacts of (A) northward and (B) southward transplants on nitrogen cycle.
**Figure S7.** Impacts of transplant on genes associated with carbon degradation.
