# Supplementary material for: Microbial responses to southward and northward Cambisol soil transplant
Source: Microbiologyopen. 2015 Oct 26;4(6):931–40. doi: 10.1002/mbo3.302 (PMC4694145; doi:10.1002/mbo3.302)
Supplement: Supplementary file 1 — Table S1. The effect of soil transplant on functional and taxonomic community composition revealed by the dissimilarity test of adonis a. Table S2. Partial Mantel tests linking environmental variables to GeoChip and DGGE data. Figure S1. Microbial biomass measured by the phospholipid fatty acid (PLFA) analysis. Figure S2. Microbial composition of (A) CN and (B) CS samples revealed by Venn diagrams. Figure S3. (A) The effect of soil transplant on microbial functional and coarse‐scale taxonomic alpha diversity in maize cropped soil. Figure S4. Multiple regression tree analysis of GeoChip data. Figure S5. Pearson correlation between dissimilarity of microbial functional compositions and environmental variables. Figure S6. Impacts of (A) northward and (B) southward transplants on nitrogen cycle. Figure S7. Impacts of transplant on genes associated with carbon degradation. [file MBO3-4-0931-s001.docx]

**Supplemental Materials**

**Table S1.** The effect of soil transplant on functional and taxonomic community composition revealed by the dissimilarity test of *adonis*^a^.

|  | GeoChip | |  | DGGE | |
| --- | --- | --- | --- | --- | --- |
|  | R^2^ | *P* |  | R^2^ | *P* |
| CC vs. CN | **0.40**^b^ | **0.07** |  | **0.93** | **0.02** |
| CC vs. CS | **0.60** | **0.03** |  | **0.96** | **0.05** |
| CN vs. CS | **0.46** | **0.01** |  | **0.90** | **0.01** |

^a^Permutational Multivariate Analysis of Variance.

^b^Significantly changed values (*P*<0.10) are shown in bold.

**Table S2.** Partial Mantel tests linking environmental variables to GeoChip and DGGE data.

| Environmental variables | GeoChip | |  | DGGE | |
| --- | --- | --- | --- | --- | --- |
|  | Statistic R | *P* |  | Statistic R | *P* |
| MAT^a^ | 0.189 | 0.139 |  | **0.567** | **0.010** |
| MAP | **0.299**^b^ | **0.064** |  | **0.375** | **0.040** |
| Relative humidity | **0.284** | **0.056** |  | **0.423** | **0.026** |
| pH | -0.269 | 0.941 |  | -0.507 | 0.993 |
| Moisture | **0.338** | **0.026** |  | **0.418** | **0.034** |
| Soil temperature | **0.260** | **0.066** |  | **0.377** | **0.028** |
| Organic matter | -0.053 | 0.499 |  | -0.075 | 0.657 |
| NO_3_^-^-N | 0.254 | 0.148 |  | **0.285** | **0.044** |
| NH_4_^+^-N | 0.107 | 0.266 |  | 0.048 | 0.310 |
| TN | 0.221 | 0.153 |  | -0.045 | 0.524 |
| TP | -0.287 | 0.934 |  | -0.289 | 0.984 |
| TK | -0.286 | 0.937 |  | -0.433 | 0.994 |
| AN | -0.369 | 0.994 |  | -0.464 | 0.997 |
| AP | -0.266 | 0.920 |  | -0.435 | 0.998 |
| AK | -0.141 | 0.795 |  | -0.124 | 0.745 |

^a^Abbreviation: MAT: mean annual temperature; MAP: mean anual precipitation; TN: total nitrogen; TP: total phosphorus; TK: total potassium; AN: alkali-hydrolyzable nitrogen; AP: available phosphorus; AK: available potassium.

^b^Significantly changed values (*P*<0.10) are shown in bold.


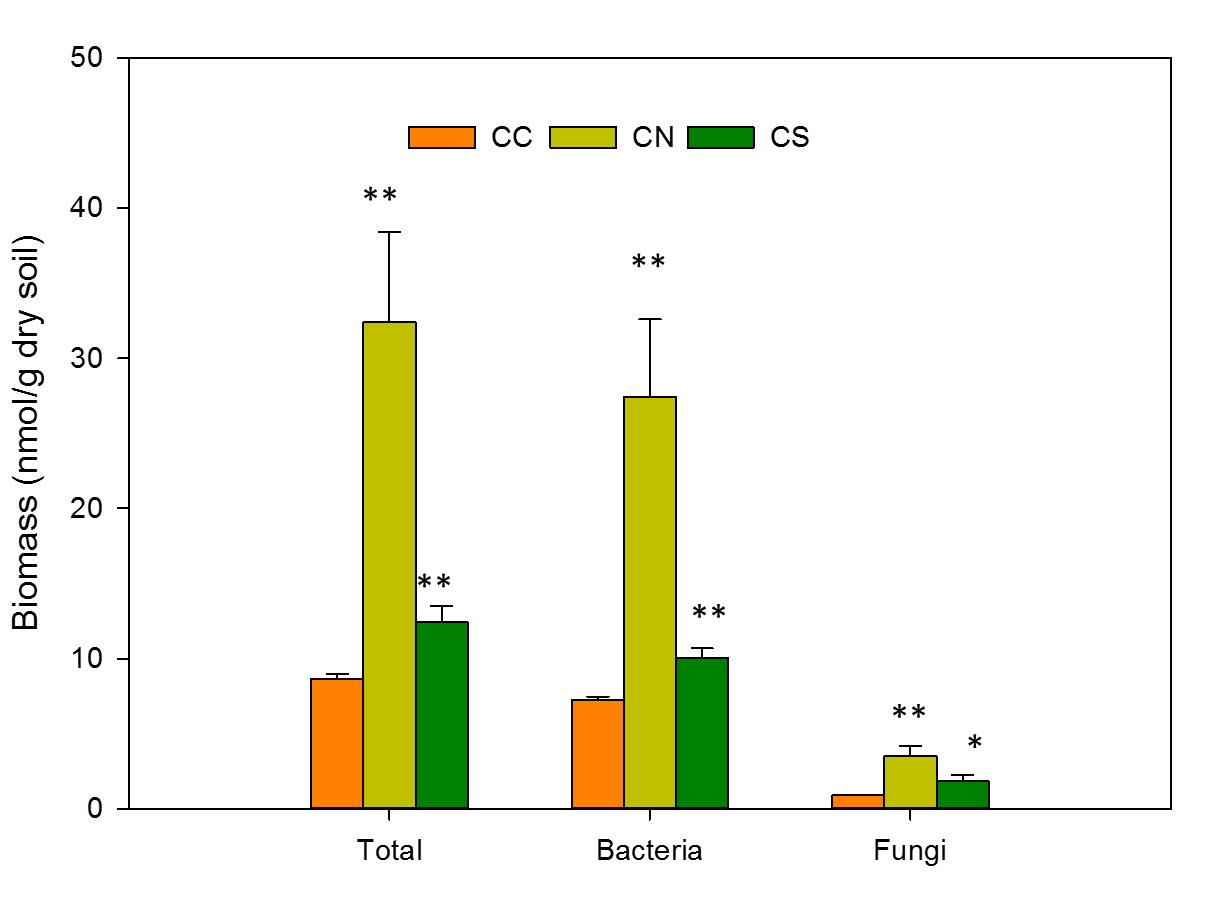


**Figure S1.** Microbial biomass measured by the phospholipid fatty acid (PLFA) analysis. The error bars represent standard errors (SE, n=3). Significant differences between CC and CN, or between CC and CS, were examined by two-tailed *t*-tests with equal variances. Significance is indicated by ‘*’ for *P* <0.10 or ‘**’ for *P* < 0.05.

A B

**
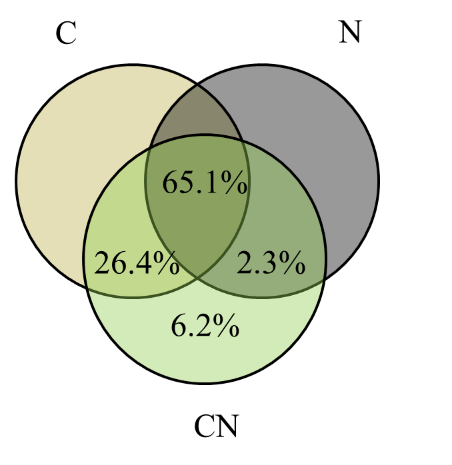

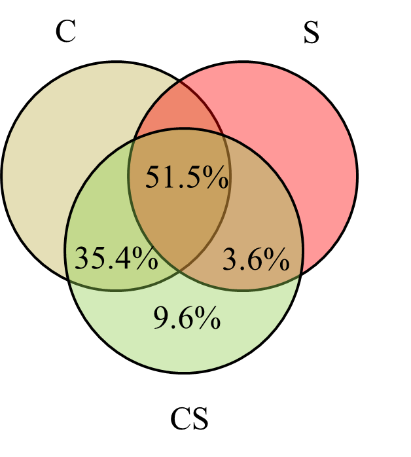
**

**Figure S2.** Microbial compostion of (A) CN and (B) CS samples revealed by Venn diagrams. Detected genes of transplanted samples were compared with donor or destination samples. Donor samples were collected in Fengqiu (C), and destination samples were collected in Hailun (N) and in Yingtan (S), respectively.

A


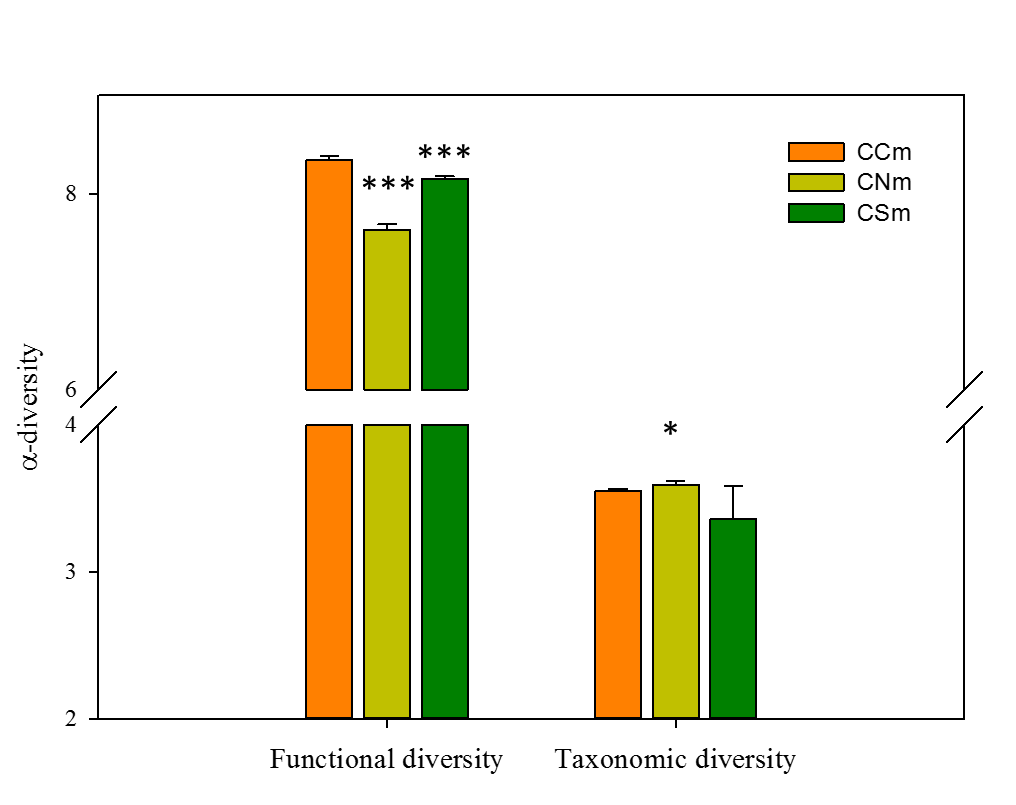


B

**Figure S3.** (A) The effect of soil transplant on microbial functional and coarse-scale taxonomic alpha-diversity in maize cropped soil. Significant differences between CC and CN, or between CC and CS, were tested by two-tailed *t*-tests with equal variances, as indicated by ‘**’ for *P* < 0.05 or ‘***’ for *P* < 0.001. (B) Pearson correlation between microbial functional and coarse-scale taxonomic alpha-diversity in maize cropped soil.


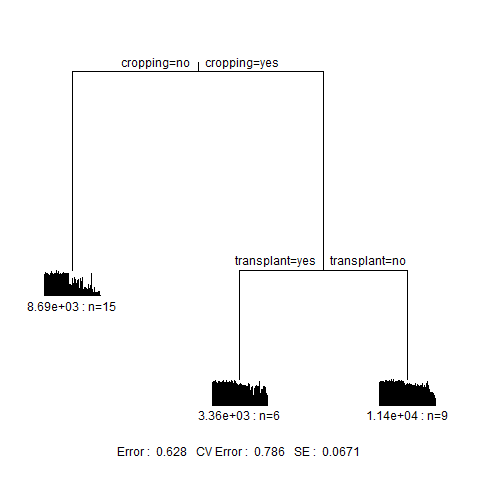


**Figure S4.** Multiple regression tree analysis of GeoChip data.

**Figure S5.** Pearson correlation between dissimilarity of microbial functional compositions and dissimilarity of environmental variables. The environmental variables matrix include MAT (mean annual temperature), MAP (mean anual precipitation), relative humidity, soil moisture, soil temperature, NO_3_^-^-N, organic matter, pH, and nitrification capacity, which were selected by CCA models or significantly changed (*P*<0.05) by soil transplant.

A


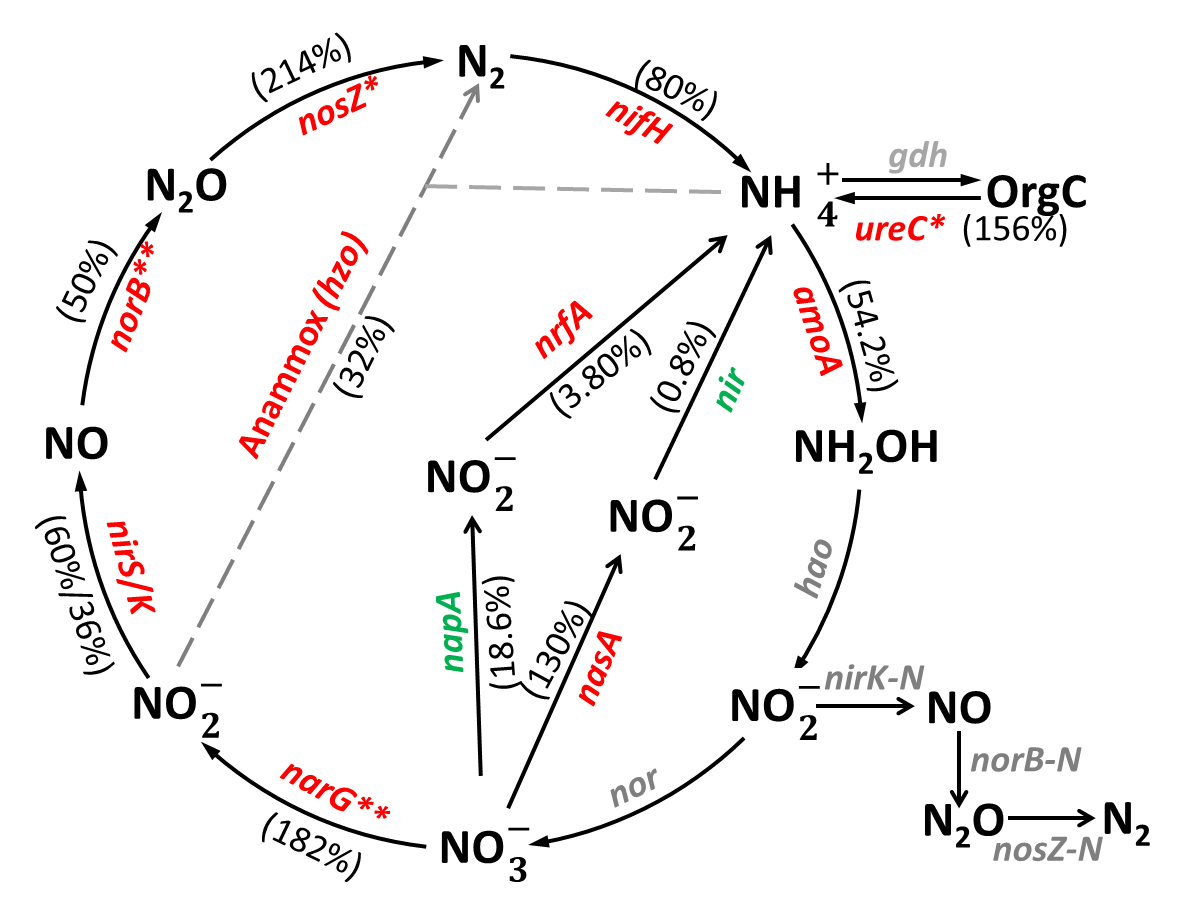


B


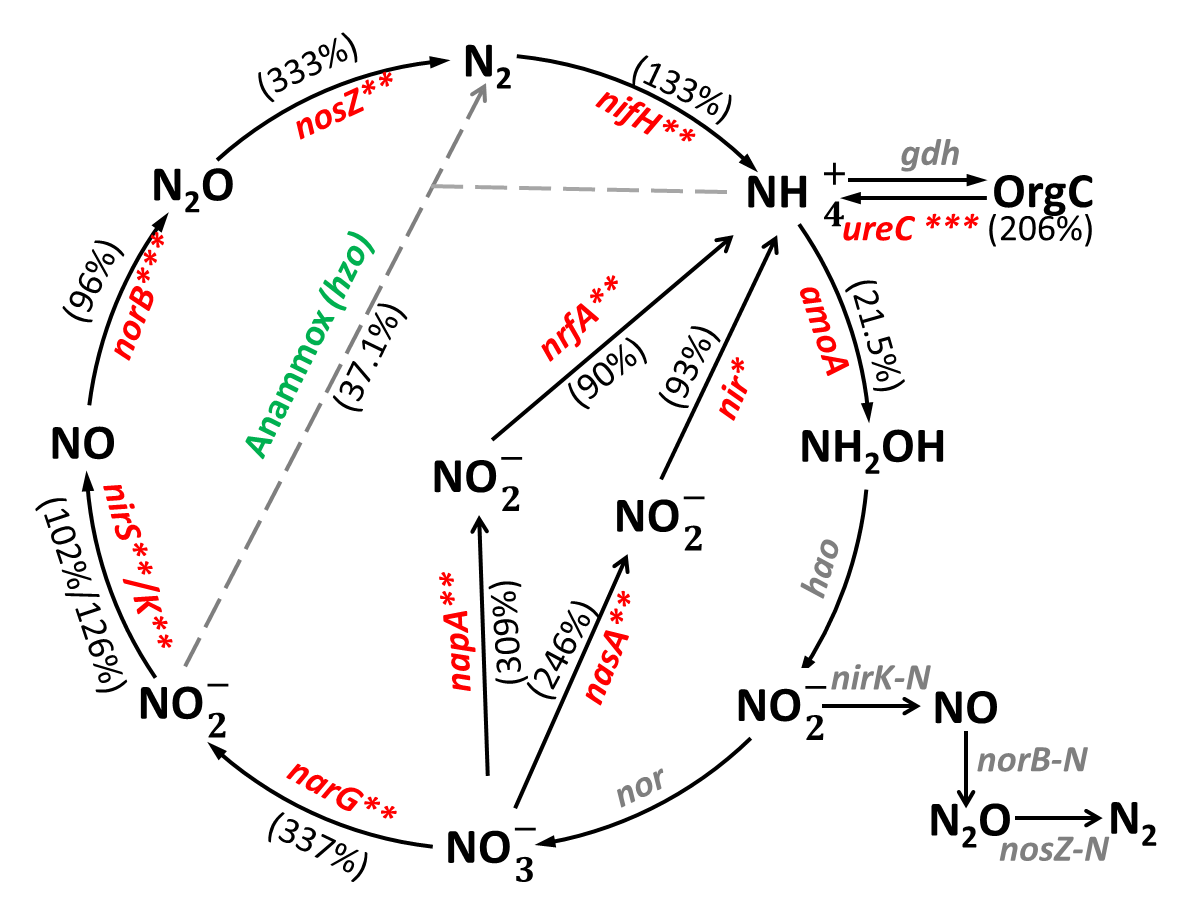


**Figure S6.** Impacts of (A) northward and (B) southward transplant on nitrogen cycle. The percentages in brackets were calculated by dividing total signal intensities of each gene in transplanted samples (CN, CS) by those in control samples (CC) and then subtracting one. Red or green genes represent genes that incraesed or decreased at transplanted sites, respectively. Grey genes were undetected by GeoChip 3.0. Significant differences between CC and CN or CC and CS were examined by two-tailed *t*-tests, indicated by ‘**’ for *P*<0.05 and ‘*’ for *P*<0.10.

**
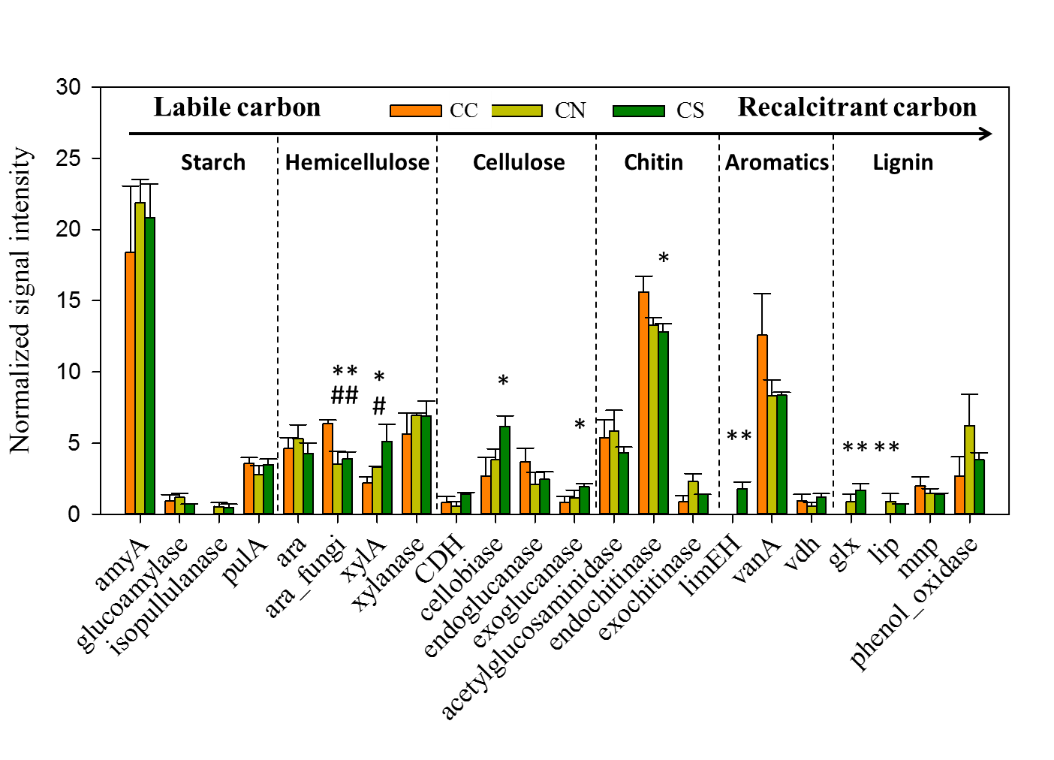
**

**Figure S7.** Impacts of transplant on genes associated with carbon degradation. Normalized signal intensities are average abundances of detected genes. Signficant differences were tested by two-tailed *t*-tests, indicated by ‘#’ between CC and CN and ‘*’ between CC and CS. ‘##’ or ‘**’ indicates *P*<0.05 and ‘#’ or ‘*’ indicates *P*<0.10.
